# RNA-Sequencing Analysis of the Spleen and Gill of *Takifugu rubripes* in Response to *Vibrio harveyi* Infection

**DOI:** 10.3389/fvets.2021.813988

**Published:** 2022-01-31

**Authors:** Dongxu Gao, Wei Lei, Chenshi Wang, Ping Ni, Xiaoyu Cui, Xindi Huang, Shigen Ye

**Affiliations:** ^1^Key Laboratory of Mariculture and Stock Enhancement in North China's Sea, Ministry of Agriculture and Rural Affairs, College of Fisheries and Life Science, Dalian Ocean University, Dalian, China; ^2^State Environmental Protection Key Laboratory of Marine Ecosystem Restoration, National Marine Environmental Monitoring Center, Dalian, China

**Keywords:** *Takifugu rubripes*, *Vibrio harveyi*, RNA-sequencing, immune response, aquacultural species

## Abstract

*Takifugu rubripes* is commonly subjected to the disease-causing bacterium, *Vibrio harveyi*. However, the mechanism involved in the immune response of *T. rubripes* to *V. harveyi* infection is unclear. We conducted a transcriptomic analysis of the spleen and gill from *T. rubripes* infected with *V. harveyi*. We obtained 60,981,357 and 60,760,550 clean reads from the control and infected spleens, and 57,407,586 and 57,536,651 clean reads from the control and infected gills, respectively. We also identified 1,560 and 1,213 differentially expressed genes in the spleen and gill, respectively. Gene ontology analysis revealed that the most enriched biological process in both the spleen and gill was “immune response”. The most enriched Kyoto Encyclopedia of Genes and Genomes immune response–related pathways were the NOD-like receptor signaling pathway in the spleen and cytokine–cytokine receptor interaction in the gill. We found 10 candidate immune-related genes in the spleen and gill. These putative immune pathways and candidate genes will provide insight into the immune response mechanisms of *T. rubripes* against *V. harveyi*.

## Introduction

*Takifugu rubripes* is becoming one of the most economic aquatic fish species in East Asia (1–3). The total aquacultural yield of *T. rubripes* in China reached 17,473 tons in 2019 (4). In Japan, these aquacultural species is considered one of the most valuable commercial finfish in recent decades (5). However, the aquacultural industry for *T. rubripes* is restricted by several serious aquatic diseases (6, 7). Specifically, high mortality resulting from *Vibrio harveyi* infections leads to enormous economic losses (8, 9). *V. harveyi* is an important luminous marine bacterium (10, 11) that is pathogenic to many aquatic animals (12, 13).

Fish immunology has received much attention for its important and unique role in understanding the evolution of immune system. Investigating the effects of bacterial infections on fish immune organs is important for understanding the immune response mechanisms to bacterial diseases (14, 15). The spleen and gill are important immune organs in fish. The spleen is the primary hematopoietic and peripheral lymphoid organ (16, 17) and is important for antigen (e.g., bacteria) presentation and initiation of adaptive immune responses (18, 19). The gill is a type of mucosal surface and a mucosal immune organ in fish (14, 20), and is an important site of bacterial exposure and host defense mechanisms (14).

Sequencing technology is widely applied in aquaculture (21–24). RNA-sequencing technology can effectively reveal genes that are engaged in immune responses and expressed in response to the presence of toxicants or infection (25–27). Many studies have focused on the transcriptomic changes in different fish tissues after bacterial infection (15, 28). However, few studies have reported the combined analysis of RNA-sequencing in the spleen and gill of *T. rubripes* after *V. harveyi* infection.

Here, we used RNA-sequencing technology to detect genome-wide transcriptional changes in the spleen and gill of *V. harveyi*–infected *T. rubripes*. These results may help identify the immune-relevant genes and mechanisms during *V. harveyi* infection. Our study provides a novel strategy for understanding the mechanisms of action of *V. harveyi*–induced aquacultural diseases in fish and developing genetic markers for *V. harveyi* disease resistance.

## Materials and Methods

### Experimental Animals and Tissue Collection

The Animal Care and Use Committee of the Key Laboratory of Mariculture and Stock Enhancement in North China's Sea at Dalian Ocean University approved all fish-related procedures in this study. *T. rubripes* (weighing 118 ± 7.5 g) were obtained from a local supplier (Tianzheng Industrial, Dalian, China) and acclimated for approximately 7 days in seawater at 19 ± 1°C.

Fish were challenged in six seawater tanks with three control and three treatment groups. The identified *V. harveyi* were reisolated from a symptomatic *T. rubripes* with skin and visceral lesions. Fifteen fish were put into each tank with 2.5 × 10^7^ colony-forming units per milliliter of *V. harveyi*, exposed to the bacteria for 12 h, then transferred to clean seawater and maintained for 7 days. The same number of fish was used as controls. Fish in the control group stayed in clean seawater throughout the experiment. One-third of the seawater was replaced every 2 days throughout the experiment. On day 7 post-challenge, some fish in the treatment group showed slow movement, decreased vitality, and cell necrosis in their spleens and gills. The control fish displayed no abnormalities in their movement, vitality, or visceral organs [see more details in Supplementary Figure 1; (29)]. The spleen and gill were collected from both the symptomatic *V. harveyi*–treated fish and control fish on day 7. Samples were frozen in liquid nitrogen prior to RNA extraction.

### Library Preparation for Transcriptome Sequencing

Sequencing analysis was performed to evaluate the effects of *V. harveyi* on global transcription in the spleen and gill. In both the control and treatment groups, the fish from the three tanks were firstly mixed, and then the four fish were randomly selected from the mixed fish. The selected samples were taken for sequencing analysis. RNA-sequencing and library preparation were performed by Novo Genomic Services Lab (Qingdao, Shandong, China). RNA (3 μg per sample) was used as the input material for the RNA sample preparation. Sequencing libraries were generated using the NEBNext Ultra RNA Library Prep Kit for Illumina (NEB, Ipswich, MA, USA) per the manufacturer's recommendations, and index codes were added to attribute sequences to each sample.

The index-coded samples were clustered using a cBot Cluster Generation System with a TruSeq PE Cluster Kit v3-cBot-HS (Illumina; NEB) per the manufacturer's instructions. After cluster generation, the library preparations were sequenced on an Illumina HiSeq platform, and 125/150-bp paired-end reads were generated.

### RNA Extraction and Reverse Transcription

Total RNA was extracted from the spleens and gills using TRIzol reagent (Invitrogen, Carlsbad, CA, USA) per the manufacturer's protocol. First-strand cDNA was synthesized from 1 μg of total RNA using a MonScript RTIII All-in-One Mix kit (Monad, Shanghai, China) per the manufacturer's protocol.

### Real-Time Quantitative PCR

Real-time quantitative (RT-q) PCR was performed to validate the sequencing analysis results on a StepOnePlus Real-Time PCR system (ABI, USA) using SYBR green I fluorescent dye. Gene expression levels were normalized to *T. rubripes* β*-actin* (30). Relative gene expression was calculated using the 2^−Δ*ΔCT*^ method (31). The primer sequences were designed using software Primers Premier 5.0 (Supplementary Table 1). Eight genes were randomly selected for RT-qPCR verification.

### Data Analysis

High-quality clean reads were obtained from raw reads. The reference genome and gene model annotation files were directly downloaded from the genome website (ftp.ensembl.org/pub/release-92/fasta/takifugu_rubripes/). Hisat2 v2.0.5 was used to build the index of the reference genome and align the paired-end clean reads to the reference genome (*Takifugu_rubripes_Ensemble_92*) (32). FeatureCounts v1.5.0-p3 was used to count the read numbers mapped to each gene (33). The fragments per kilobase of transcript sequence per millions base pairs sequenced of each gene was then calculated based on the gene length, and read counts were mapped to the gene. Differential expression analysis of two conditions was performed using the DESeq2 R package (1.16.1) (34), which provides statistical routines for determining differential expression in digital gene expression data using a model based on the negative binomial distribution. The resulting *p*-values were adjusted using the Benjamini and Hochberg approach for controlling the false discovery rate. Genes with an adjusted *p*-value < 0.05 in DESeq2 were assigned as differentially expressed genes (DEGs). Gene ontology (GO) enrichment analysis of the DEGs was implemented by the clusterProfiler R package, which corrects for gene length bias. GO terms with corrected *p* < 0.05 were considered significantly enriched by DEGs (35). The Kyoto Encyclopedia of Genes and Genomes (KEGG) database enables understanding high-level functions and utilities of biological systems, such as cells, organisms, and ecosystems, from molecular-level information, especially large-scale molecular datasets generated *via* genome sequencing and other high-throughput experimental technologies (http://www.genome.jp/kegg/). We used clusterProfiler R to test the statistical enrichment of the DEGs in the KEGG pathways (36). The top GO categories and KEGG pathways were selected according to their *p*-values.

## Results

### Differential Gene Expression in the Spleen After *V. harveyi* Infection

**Figure 1:**
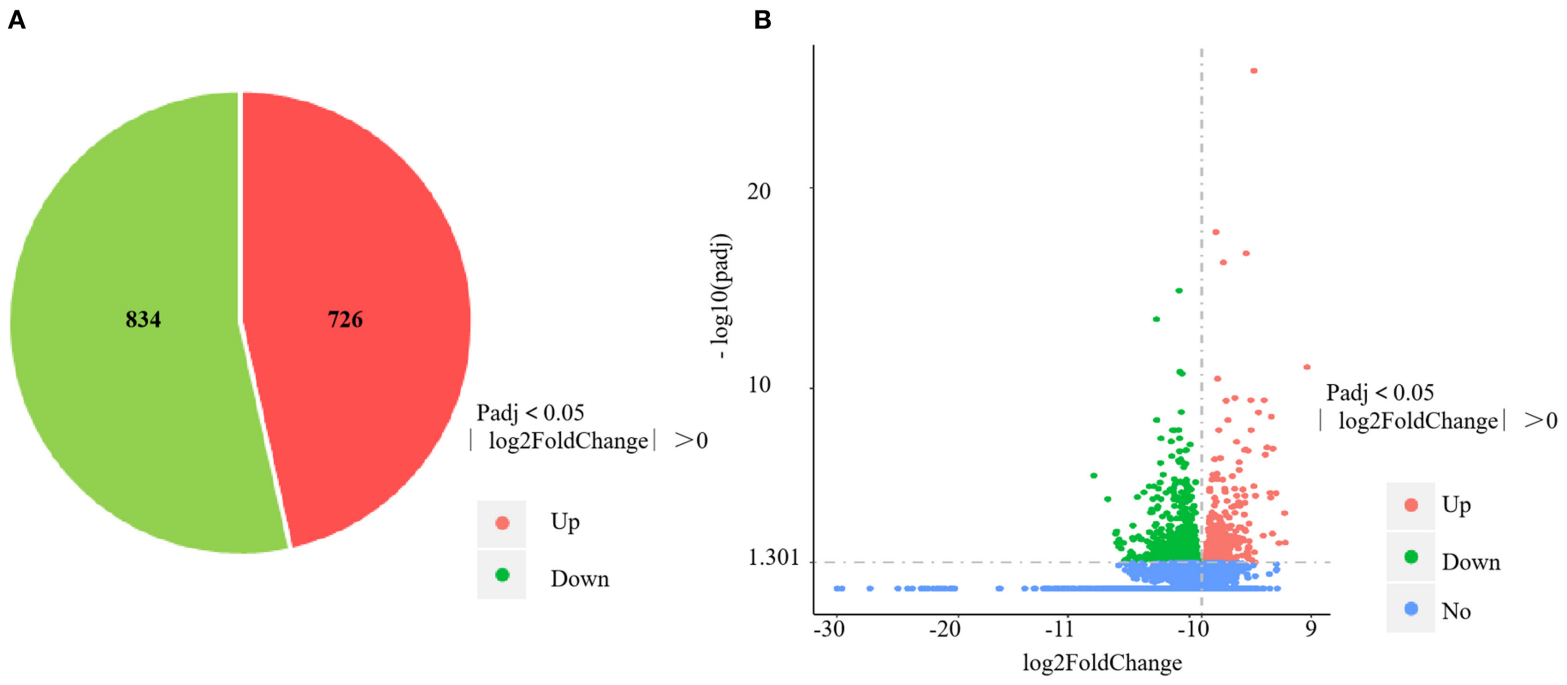
DEGs identified in the spleen infected by *V. harveyi*. The number of DEGs in spleen is shown in **(A)**, and the volcano plot of DEGs in spleen is shown in **(B)**.

The RNA-sequencing data were submitted to Gene Expression Omnibus (accession number: GSE155911). The four control spleens (CS1–4) yielded 60,298,712; 59,160,768; 61,669,660 and 62,796,286 clean reads, respectively. The four *V. harveyi*–infected spleens (VhS1–4) yielded 61,129,742; 67,177,292; 55,859,620; and 58,875,544 clean reads, respectively. The mapping rates were 88.94%, 87.47%, 88.88%, and 88.87% for the four control spleens (CS1–4), respectively. The mapping rates were 89.50%, 89.24%, 89.29%, and 89.61% for the four infected spleens (VhS1–4), respectively. Compared with the controls, the spleens of the *V. harveyi*–infected fish contained 1,560 DEGs (*p* < 0.05, fold difference >1). Of these, 726 genes were significantly upregulated, and 834 were significantly downregulated (Figure 1A). Figure 1B shows the volcano plot of the DEG distribution.

GO analysis results for the spleen tissue showed that these DEGs were clustered into predicted functional groups. The effects of *V. harveyi* were demonstrated in 1,939 groups, including 1,344 biological process (BP) terms (69.31%), 205 cellular component (CC) terms (10.57%), and 390 molecular function (MF) terms (20.12%). In the BP category, “immune response” (GO:0006955), “response to external biotic stimulus” (GO:0043207), and “regulation of immune response” (GO:0050776) were most noteworthy. The most highly represented CC term was “extracellular region” (GO:0005576). The most highly enriched MF terms were “enzyme regulator activity” (GO:0030234) and “enzyme inhibitor activity” (GO:0004857; Figure 2A).

**Figure 2:**
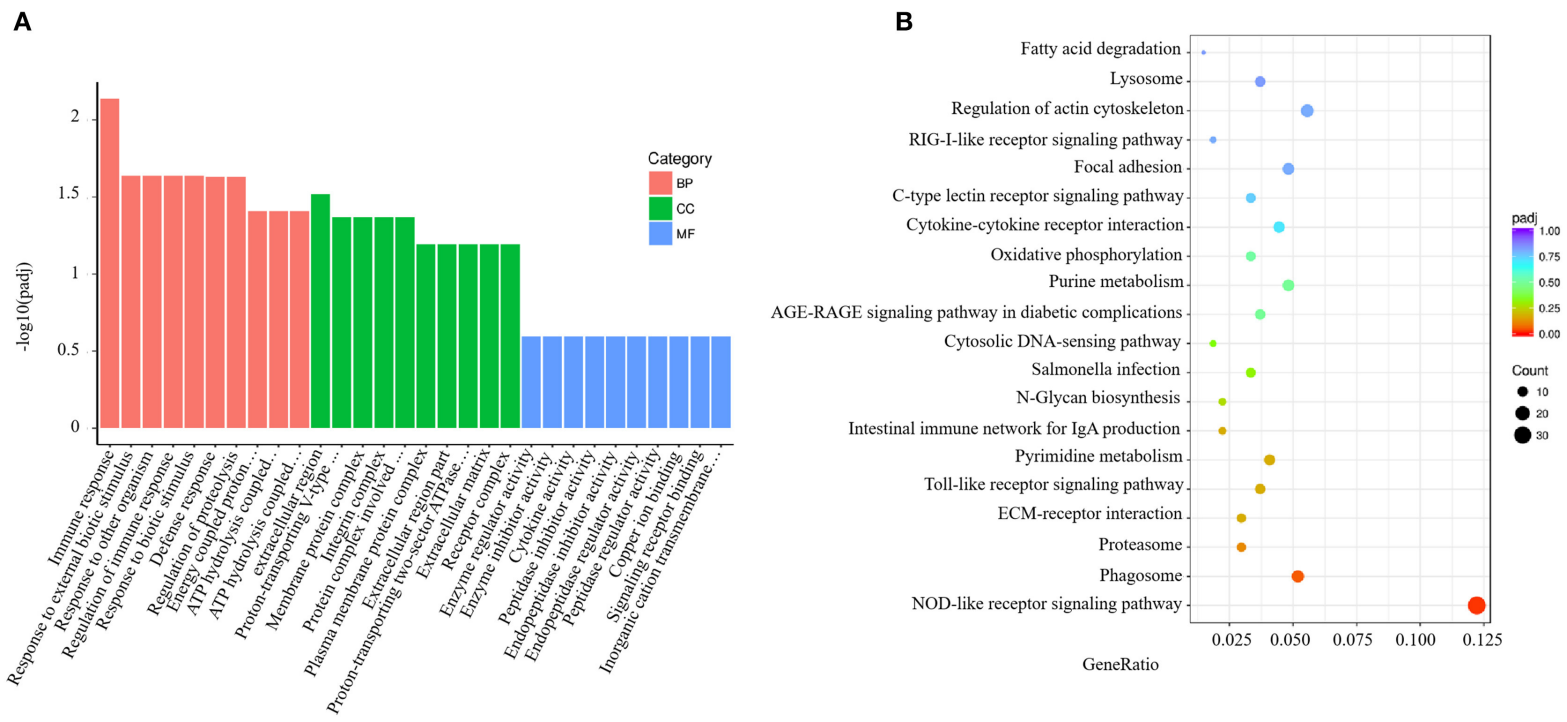
Enrichment GO and KEGG annotation in the spleen infected by *V. harveyi*. Top GO categories in the spleen are shown in **(A)**. KEGG category of DEGs in the spleen infected by *V. harveyi* is shown in **(B)**.

Using KEGG functional annotations, the 1,560 DEGs were classified to identify the pathways in which they participate. The DEGs were mapped to 115 KEGG pathways, and the top 20 most common pathways were identified, among which, the most significant and highly enriched pathway was the NOD-like receptor signaling pathway (Figure 2B).

### Differential Gene Expression in the Gill After *V. harveyi* Infection

**Figure 3:**
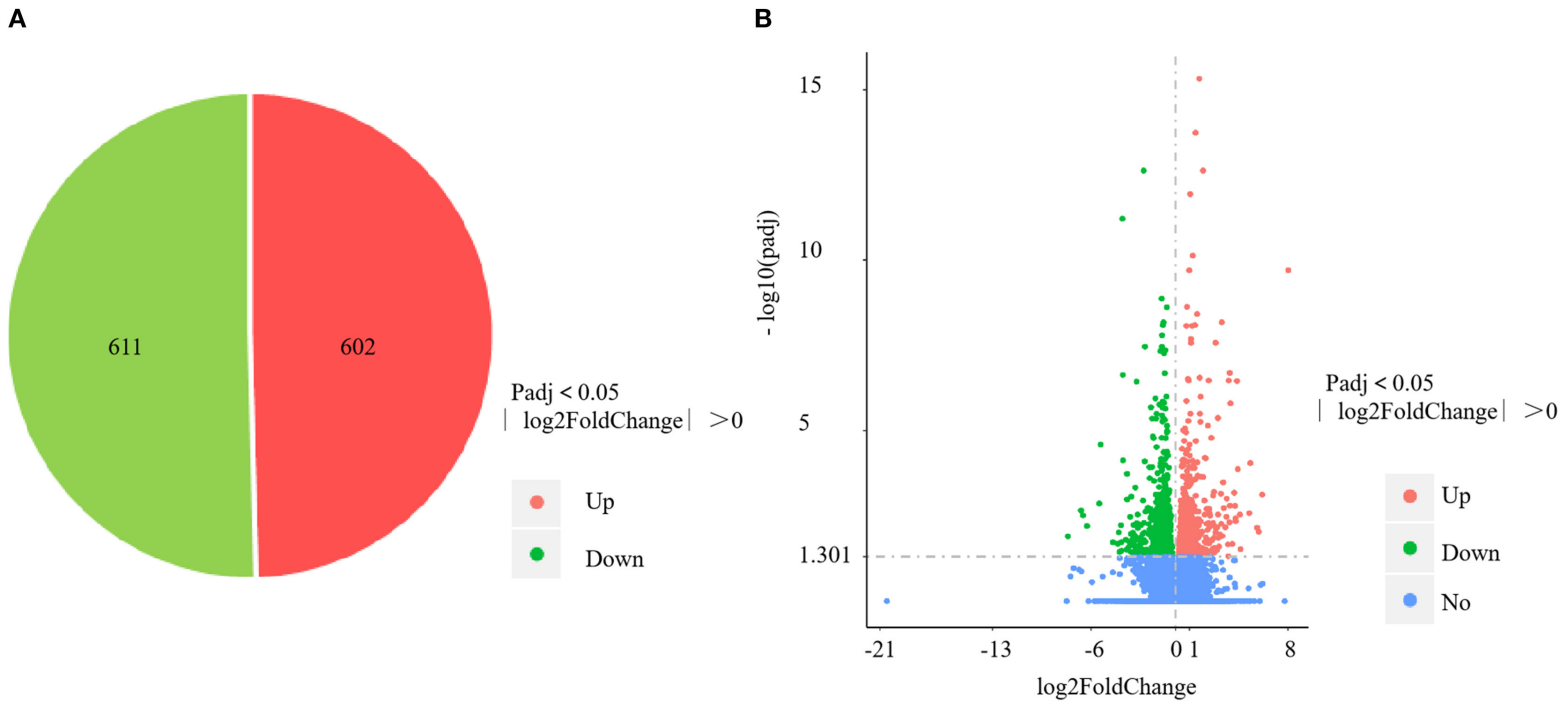
DEGs identified in the gill infected by *V. harveyi*. The number of DEGs in gill is shown in **(A)**, and the volcano plot of DEGs in gill is shown in **(B)**.

The four control gills (CG1–4) yielded 57,172,556; 58,742,866; 57,255,304; and 56,459,618 clean reads, with mapping rates of 88.04%, 88.13%, 88.65%, and 88.09%, respectively. The four *V. harveyi*–infected gills (VhG1–4) yielded 58,236,430; 63,555,364; 54,010,958; and 54,343,850 clean reads, with mapping rates of 88.34%, 88.58%, 88.69%, and 88.41%, respectively. The RNA-sequencing results yielded 1,213 DEGs, including 602 upregulated and 611 downregulated genes (*p* < 0.05, fold difference >1) in the gills after *V. harveyi* treatment relative to the controls (Figure 3A). These 1,213 genes were hierarchically clustered to produce a volcano plot (Figure 3B).

*V. harveyi* significantly altered the GO analysis results for the gills, yielding 1,743 GO terms, including 1,235 BP terms (70.85%), 159 CC terms (9.12%), and 349 MF terms (20.03%). In the GO term for BP, much more attention was paid to “immune response” (GO:0006955), “immune system process” (GO:0002376), “regulation of immune system process” (GO:0002682), “regulation of immune response” (GO:0050776), and “positive regulation of immune system process” (GO:0002684). The most enriched CC term was “integrin complex” (GO:0008305); the most enriched MF term was “extracellular matrix structural constituent” (GO:0005201; Figure 4A).

**Figure 4:**
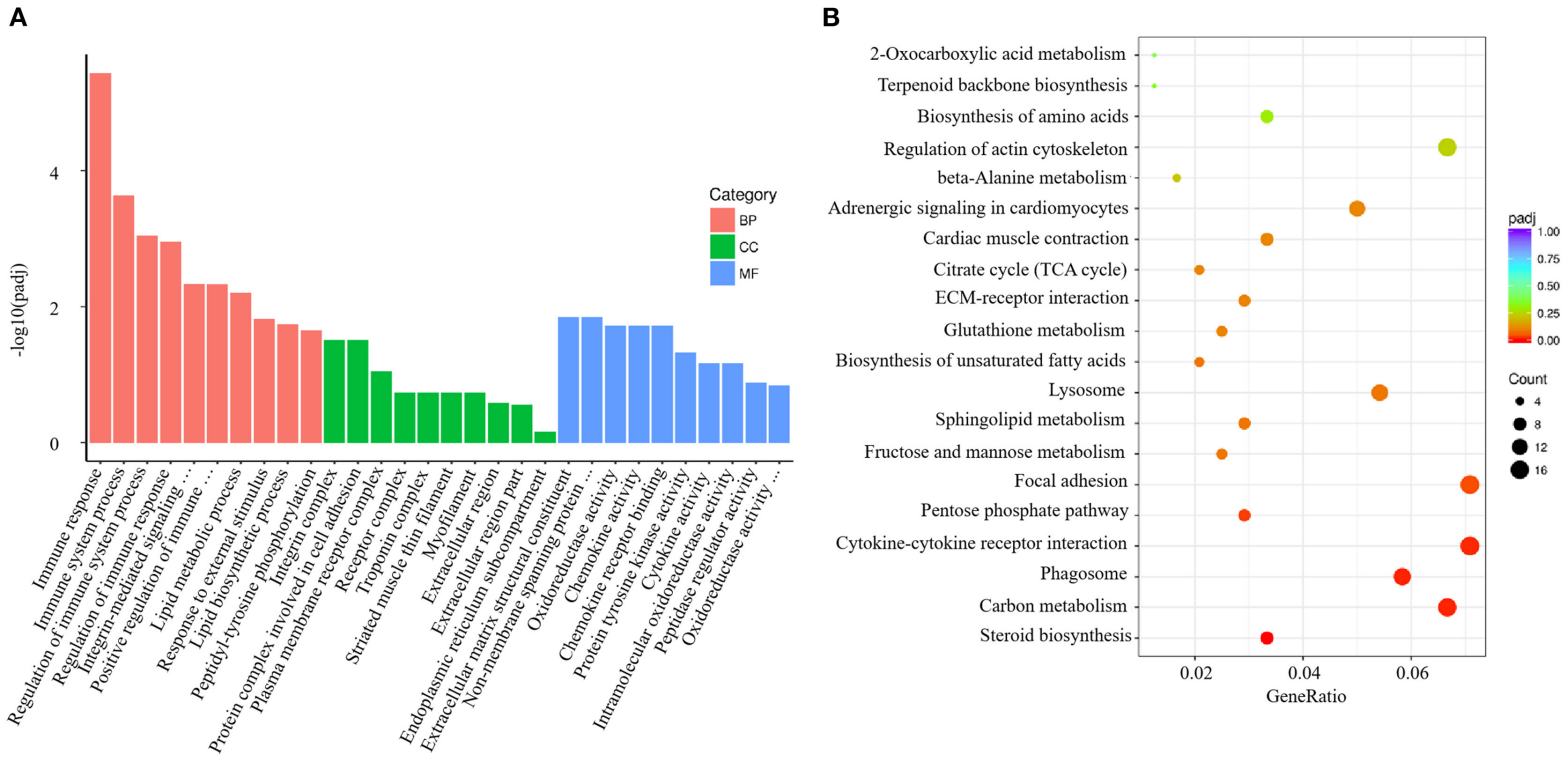
Enrichment GO and KEGG annotation in the gill infected by *V. harveyi*. Top GO categories in the gill are shown in **(A)**. KEGG category of DEGs in the gill infected by *V. harveyi* is shown in **(B)**.

In the gills, the DEGs were mapped to 116 KEGG pathways, and the top 20 representative enriched KEGG pathways were identified. Cytokine–cytokine receptor interaction, which is related to immune response, was highly enriched (Figure 4B).

### Combined RNA-Sequencing Analysis of the Spleen and Gill

**Figure 5:**
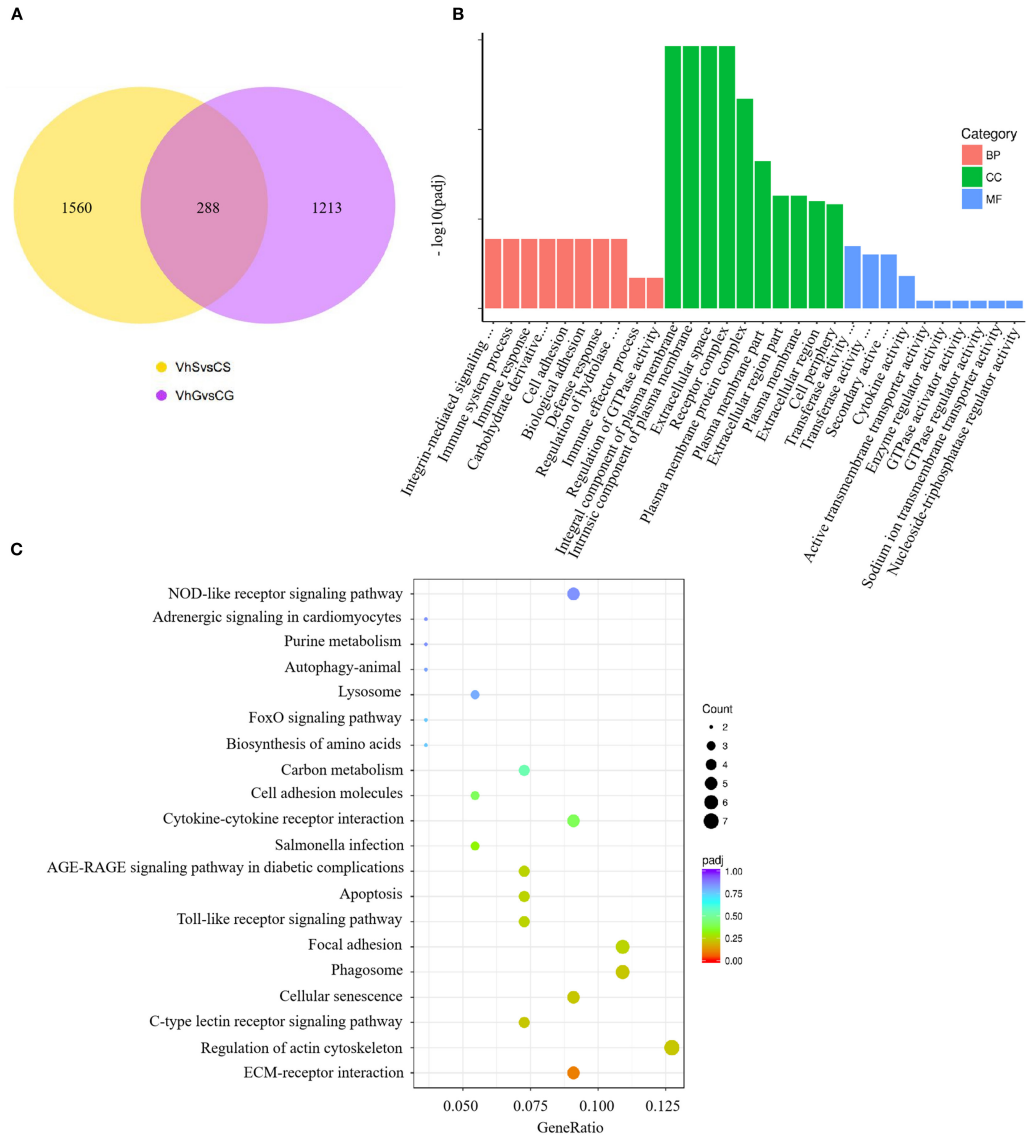
Combined RNA-sequencing analysis of the spleen and gill. The overlapping DEGs in spleen and gill are shown in **(A)**. Top GO categories of overlapping DEGs are shown in **(B)**. The KEGG analysis of overlapping DEGs is shown in **(C)**.

To investigate the effects of *V. harveyi* infection in both the spleen and gill, we constructed a Venn diagram to find the common genes from significant DEGs in the spleen and gill (with *p* < 0.05, fold change > 1). We found 288 overlapping genes in these organs (Figure 5A), which were then assigned to 619 GO terms: 413 BP terms (66.72%), 66 CC terms (10.66%), and 140 MF terms (22.62%). For the BP terms, “immune system process” (GO:0002376), “immune response” (GO:0006955), and “immune effector process” (GO:0002252) were highly enriched. “Integral component of plasma membrane” (GO:0005887) was the most significantly enriched CC term, and “transferase activity, transferring glycosyl groups” (GO:0016757) was the most significantly enriched MF term (Figure 5B). Overlapping DEGs were mapped to 29 KEGG pathways. In the top 20 representative enriched KEGG pathways, much more attention was paid to the C-type lectin receptor signaling pathway and the Cellular senescence which were related to immune response (Figure 5C). These findings indicate that *V. harveyi* infection could lead to abnormal gene expression and trigger immune responses in both the spleen and gill.

To better understand the mechanisms of action of *V. harveyi*–induced disease in *T. rubripes*, we analyzed 23 immune-related DEGs from our transcriptomic dataset of the spleen and gill. Ten of these 23 DEGs were found in both the spleen and gill (Table 1).

**Table 1:** Partial differentially expressed immune-related genes in *T. rubripes* after *V. harveyi* infection.

	**Gene catalog**	**Organ**	**Fold change**
Interleukin	Interleukin-1b	Spleen	4.05
	interleukin-2	Spleen	−2.43
	interleukin-6	Spleen/gill	2.44/5.16
	Interleukin-8	Spleen/gill	2.37/6.39
	Interleukin-16	Gill	−1.33
	interleukin-21	Gill	−1.93
Complement component	complement component 7a	Spleen/gill	−1.97/−1.96
	complement component 7b	Spleen/gill	34.82/258.31
	complement component 6	Gill	3.38
Toll-like receptor	toll-like receptor 5	Spleen	−3.83
	toll-like receptor 7	Spleen/gill	−1.97/−1.43
	toll-like receptor 2	Gill	−1.66
Interferon regulatory factor	interferon regulatory factor 7	Spleen	−2.09
	interferon regulatory factor 1b	Spleen/gill	−1.59/−1.53
	interferon regulatory factor 8	Gill	−1.37
Other genes related to immune response	NK-lysin tandem duplicate 4	Spleen/gill	−2.79/−1.94
	carnitine palmitoyltransferase 1B (muscle)	Spleen	2.09
	isocitrate dehydrogenase 1	Gill	1.35
	coagulation factor II (thrombin) receptor	Spleen/gill	2.09
	transcription factor 7	Spleen/gill	−1.68
	tryptophan hydroxylase 1	Gill	3.25
	wingless-type MMTV integration site family, member 4a	Gill	−2.10
	SATB homeobox 1b	Spleen/gill	−2.32

### DEG Validation *via* RT-qPCR

**Figure 6:**
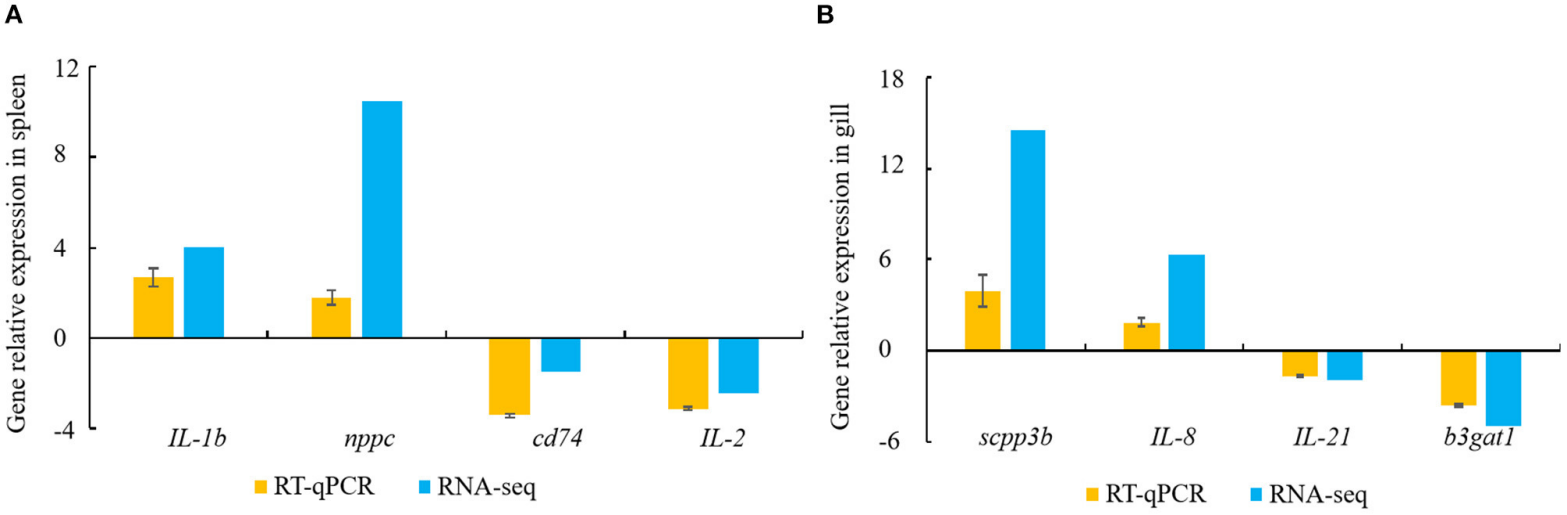
DEGs validated by RT-qPCR. **(A)** spleen; **(B)** gill. Gene expression analysis from RNA-sequencing results and RT-qPCR validation results (*n* = 4).

Constitutive changes in the DEGs identified *via* RNA-sequencing were consistent with the RT-qPCR results from the spleen and gill samples. The RNA-sequencing data for the spleen showed that *V. harveyi* infection significantly upregulated the expressions of *IL-1b* (by 4.05-fold) and *nppc* (by 10.46-fold) compared with those of the controls. The expression changes of *IL-1b* (by 2.68-fold) and *nppc* (by 1.81-fold) were confirmed *via* RT-qPCR (Figure 6A). The significantly downregulated genes, *cd74* (by 1.48-fold) and *IL-2* (by 2.43-fold), were also validated *via* RT-qPCR (downregulated by 3.42- and 3.12-fold, respectively; Figure 6A). The RNA-sequencing data for the gill showed that *V. harveyi* infection significantly upregulated the expressions of *scpp3b* (by 14.54-fold) and *IL-8* (by 6.39-fold) compared with those of the controls. The expression changes in *scpp3b* (by 3.91-fold) and *IL-8* (by 1.89-fold) were confirmed *via* RT-qPCR (Figure 6B). The significantly downregulated genes, *IL-21* (by 1.93-fold) and *b3gat1* (by 5.02-fold), were also validated *via* RT-qPCR (downregulated by 1.68- and 3.60-fold, respectively; Figure 6B).

## Discussion

*T. rubripes* is becoming a very important economic aquacultural species. Large-scale breeding of *T. rubripes* can easily result in disease outbreaks, which would thus reduce the food quality and economic benefits. Therefore, researchers should determine the molecular mechanisms of disease resistance in *T. rubripes*. Here, we performed RNA-sequencing analysis of *T. rubripes* spleen and gill responding to *V. harveyi* infection. Peng et al. (30) demonstrated that *V. harveyi* can alter the splenic transcriptome of *T. rubripes*; however, the effect of *V. harveyi* on the *T. rubripes* gill transcriptome remains unknown. Our study is the first to report the changes in the *T. rubripes* gill transcriptome after *V. harveyi* infection. The results of this study enrich our knowledge of the *T. rubripes* transcriptome.

Several studies have identified immune-related genes in *T. rubripes* spleen and gill (6, 37). However, few studies have reported combined analysis of immune-related DEGs in *T. rubripes* spleen and gill after *V. harveyi* infection. Our analysis yielded 1,560 and 1,213 DEGs in the spleen and gill, respectively. We performed functional enrichment analysis to further study the role of DEGs in immune-related disorders. GO and KEGG pathway analyses showed that many immune-related terms and pathways were highly enriched in the spleen and gill (Figures 2, 4, 5). To determine the common GO terms, KEGG pathways and target DEGs in the spleen and gill after *V. harveyi* infection, we conducted the first reported combined analysis of the transcriptomic changes in the spleen and gill. In the common GO category, three immune-related BP terms were highly enriched: immune system process, immune response, and immune effector process (Figure 5B). Two immune-related pathways were significantly enriched among the common KEGG annotations.

The GO and KEGG analyses revealed several important immune-related genes in the transcriptome, including genes for interleukin (IL), complement components, toll-like receptors (TLRs), interferon regulatory factors (IRFs), and others (Table 1). *IL-6, IL-8, c7a, c7b, tlr7, irf1b, NK-lysin tandem duplicate 4, coagulation factor II (thrombin) receptor, transcription factor 7*, and *SATB homeobox 1b* were differentially expressed in both the spleen and gill. Of these, *IL-6, IL-8, c7a, c7b, tlr7*, and *irf1b* caught our attention.

IL is an important cytokine involved in inflammatory and immune responses. *IL-6* is among the most important multifunctional cytokines owing to its essential roles in both innate and adaptive immune responses, and in defending against pathogenic microbial invasion (38, 39). *IL-8* plays a key role in the inflammatory responses toward bacterial infections in some fish [e.g., *Cynoglossus semilaevis* (40), *Ictalurus punctatus* (41), and *Siniperca chuatsi* (42)]. RNA-sequencing analysis results suggested that the *IL-6* and *IL-8* expression levels were highly upregulated after *V. harveyi* infection in both the spleen and gill, indicating that *IL-6* and *IL-8* are involved in anti-*V. harveyi* defenses. The complement system, activated by bacteria, is part of the innate immune system and can be recruited and activated by the adaptive immune system (26, 43). Complement component 7 (c7) plays a significant role in assembling the cytolytically active membrane attack complex within target cell membranes and performs its main function in host defenses against pathogens and promoting inflammation (44, 45). Although the complement system has been studied extensively in mammals, considerably less is known about complement in teleost fish (45–47). In addition, the functions of *c7a* and *c7b* (*c7* subtypes) in teleosts remain unclear, particularly in *T. rubripes* (48–50). Our data revealed that *c7a* was significantly downregulated, and *c7b* was significantly upregulated in both the spleen and gill. *c7a* and *c7b* were differentially expressed suggesting that the complement system might play an important role in response to *V. harveyi* infection. Why these two complement components were differentially altered remains uncertain. However, our findings may help reveal the molecular function of *c7*. TLRs are a group of pattern-recognition receptors in the innate immune system (51). Here, we identified DEGs mapped to the TLR signaling pathway, including *tlr7* in both the spleen and gill. *tlr7*, a member of the TLR family, plays an essential role in fish antibacterial immunity (52). Here, *tlr7* was significantly downregulated in both the spleen and gill, implying that innate immune genes could be altered at 7 days after *V. harveyi* infection. IRFs mediate host responses against pathogen infection and other important biological processes. Zhan et al. (53) showed that *irf1* plays an important role in defending blunt snout bream against *Aeromonas hydrophila* infection. Here, *irf1b* expression was downregulated after *V. harveyi* challenge in the spleen and gill, indicating that *irf1b* is involved in *V. harveyi–*induced immune regulation.

In this study, we performed the first reported combined RNA-sequencing analysis of the spleen and gill in *T. rubripes* infected with *V. harveyi* and screened many immune-related DEGs, GO terms, and KEGG pathways. Several immune-related genes were altered in both the spleen and gill and might play important roles in the immune response of *T. rubripes* to *V. harveyi* infection. Our results provide an important basis for further studies on the mechanisms of action of *V. harveyi*–induced aquacultural fish disease and enable better understanding this severe disease.

## Data Availability Statement

The datasets presented in this study can be found in online repositories. The names of the repository/repositories and accession number(s) can be found at: https://www.ncbi.nlm.nih.gov/geo/, GSE155911.

## Ethics Statement

The animal study was reviewed and approved by all procedures of fish used during this study were approved by the Animal Care and Use Committee of Key Laboratory of Mariculture and Stock Enhancement in North China's Sea at Dalian Ocean University.

## Author Contributions

DG, WL, and SY conceived the whole project. CW and PN carried out the animal preparation and participated in the bioinformatics analysis. XC and XH participated in the RNA extraction, reverse transcription, and real-time quantitative PCR. DG and WL wrote the article and all authors participated in the discussion. All authors approved the final article.

## Funding

This work was funded by the National Natural Science Foundation of China (Grant Nos. 42006126 and 41806187), the Doctoral Start-up Foundations of both Liaoning Province (20180540023), Dalian Ocean University (HDYJ201809), and the Major Special Project of Science and Technology of Liaoning Province (2020JH1/10200002).

## Conflict of Interest

The authors declare that the research was conducted in the absence of any commercial or financial relationships that could be construed as a potential conflict of interest.

## Publisher's Note

All claims expressed in this article are solely those of the authors and do not necessarily represent those of their affiliated organizations, or those of the publisher, the editors and the reviewers. Any product that may be evaluated in this article, or claim that may be made by its manufacturer, is not guaranteed or endorsed by the publisher.
