# Supplementary material for: RNA-Sequencing Analysis of the Spleen and Gill of Takifugu rubripes in Response to Vibrio harveyi Infection
Source: Front Vet Sci. 2022 Jan 31;8:813988. doi: 10.3389/fvets.2021.813988 (PMC8841829; doi:10.3389/fvets.2021.813988)
Supplement: Supplementary file 1 [file Data_Sheet_1.docx]

RNA-sequencing analysis of the spleen and gill of *Takifugu rubripes* in response to *Vibrio harveyi* infection

Supplementary Data


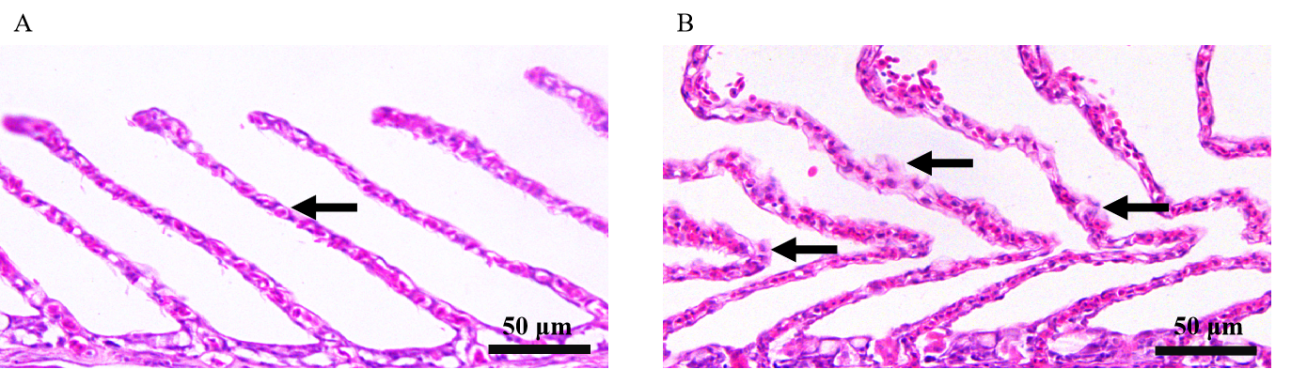


**Supplementary Figure 1.** Pathological changes of the gill of *Takifugu rubripes* in the control group (A) and the treatment group (B). The gill filaments in the control group were intac. The gill filaments in the treatment group were [necrotic](D:/%E5%BA%94%E7%94%A8%E8%BD%AF%E4%BB%B6/%E6%9C%89%E9%81%93/Dict/8.10.3.0/resultui/html/index.html#/javascript:;), that is, the gill lamellae respiratory epithelial cells had swelled and fallen off. Gill lamellae respiratory epithelial cells are indicated by the arrows.

**Supplementary Table 1.** List of primers used for RT-qPCR

| Target gene | Primer sequence |
| --- | --- |
| *IL-1b* | F: 5’- GGGAGGCTCGGAATGTCG -3’  R: 5’- TGTTGAAGAGAAAGCGAACCAGT -3’ |
| *nppc* | F: 5’- TTTAGTAGCGTGTGGACTTATGATC -3’  R: 5’- CCGGGTGTCCATGCGTA -3’ |
| *cd74* | F: 5’- TGCCAAGACCAAATGCCAG -3’  R: 5’- CCATAAGAGGCTGCTTTCGG -3’ |
| *IL-2* | F: 5’- GCGAGGATGTGAAATGTGAACC -3’  R: 5’- GTCCAGAAAGTTGGTGAGAGGCT -3’ |
| *scpp3b* | F: 5’- AGGTACTCATCAACCAGCTGCTC -3’  R: 5’- AGCAGGGATGGTGGTGGTC -3’ |
| *IL-8* | F: 5’- CACATCTCTCGTGGTGCTCC -3’  R: 5’- GGAGCTTTAGGATCCAGGCA -3’ |
| *IL-21* | F: 5’- GACAAAGTGAAGCAGCAGAGGA -3’  R: 5’- AGGCTGAAGATCTCTTTGAAATTTT -3’ |
| *b3gat1* | F: 5’- GCAACTCTTCTGTCTTCCCTACTGA -3’  R: 5’- TTGACCTTGGGGGACTCGTA -3’ |
| *β-actin*1 | F: 5’- ATCCGTAAGGACCTGTATGC-3’  R: 5’- AGTATTTACGCTCAGGTGGG -3’ |

References

1. Peng HY, Yang BX, Li BY, Cai ZL, Cui QJ, Chen MK, et al. Comparative transcriptomic analysis reveals the gene expression profiles in the liver and spleen of Japanese pufferfish (*Takifugu rubripes*) in response to *Vibrio harveyi* infection. Fish Shellfish Immunol. (2019) 90:308-16. doi: 10.1016/j.fsi.2019.04.304
